# Supplementary material for: A polygenic risk score for the QT interval is an independent predictor of drug-induced QT prolongation
Source: PLoS One. 2024 Jun 17;19(6):e0303261. doi: 10.1371/journal.pone.0303261 (PMC11182491; doi:10.1371/journal.pone.0303261)
Supplement: S1 File — (DOCX) [file pone.0303261.s001.docx]

**Supplemental Table 1. Unadjusted association with diLQTS by normalized PGS.** Shown is mean±standard deviation. After Bonferroni correction, only QT interval PGS had statistically significant association at P < 0.0025.

| **PGS** | **Cases (N = 281)** | **Controls (N = 2219)** | **F-score (df = 1)** | **P-value** |
| --- | --- | --- | --- | --- |
| QT interval^1^ | 0.2124±0.9541 | -0.0269±1.0027 | 14.36 | 0.0002 |
| High-density lipoprotein cholesterol (HDL)^2^ | -0.1567±0.9612 | 0.0198±1.0033 | 7.8 | 0.0053 |
| Apolipoprotein A^2^ | -0.1409±0.9808 | 0.0178±1.0012 | 6.3 | 0.0122 |
| Systolic blood pressure^3^ | 0.1271±1.0285 | -0.0161±0.9954 | 5.12 | 0.0237 |
| Diastolic blood pressure^3^ | 0.1247±0.9809 | -0.0158±1.0015 | 4.93 | 0.0265 |
| Abdominal aortic aneurysm^4^ | 0.1161±1.0307 | -0.0147±0.9953 | 4.27 | 0.0388 |
| Apolipoprotein B^2^ | 0.1102±0.967 | -0.014±1.0034 | 3.85 | 0.0498 |
| Low-density lipoprotein cholesterol (LDL)^2^ | 0.1037±0.946 | -0.0131±1.0061 | 3.41 | 0.0649 |
| C-reactive protein^2^ | 0.0836±0.9324 | -0.0106±1.0079 | 2.22 | 0.1368 |
| Hypertension^2^ | 0.0804±1.0627 | -0.0102±0.9916 | 2.05 | 0.1525 |
| Dilated cardiomyopathy^5^ | -0.0697±0.9979 | 0.0088±1.0001 | 1.54 | 0.2148 |
| Myocardial infarction^2^ | 0.0668±1.0337 | -0.0085±0.9956 | 1.41 | 0.2347 |
| Total cholesterol^2^ | 0.0629±0.9541 | -0.008±1.0056 | 1.25 | 0.2631 |
| Atrial fibrillation^6^ | 0.0468±1.0345 | -0.0059±0.9956 | 0.69 | 0.405 |
| Triglycerides^2^ | 0.0392±0.976 | -0.005±1.0031 | 0.49 | 0.4858 |
| Heart failure^2^ | 0.0293±1.0295 | -0.0037±0.9964 | 0.27 | 0.6018 |
| Lipoprotein(a)^3^ | -0.0245±1.0501 | 0.0031±0.9937 | 0.19 | 0.6624 |
| Ischemic stroke^7^ | -0.0201±0.9231 | 0.0025±1.0095 | 0.13 | 0.7213 |
| Coronary artery disease^8^ | -0.0078±1.0106 | 0.001±0.9989 | 0.02 | 0.89 |
| Venous thromboembolism^9^ | 0.0052±0.9847 | -0.0007±1.0021 | 0.01 | 0.9256 |
| **References**  **1.** Arking DE, Pulit SL, Crotti L, van der Harst P, Munroe PB, Koopmann TT, Sotoodehnia N, Rossin EJ, Morley M, Wang X, Johnson AD, Lundby A, Gudbjartsson DF, Noseworthy PA, Eijgelsheim M, Bradford Y, Tarasov KV, Dorr M, Muller-Nurasyid M, Lahtinen AM, Nolte IM, Smith AV, Bis JC, Isaacs A, Newhouse SJ, Evans DS, Post WS, Waggott D, Lyytikainen LP, Hicks AA, Eisele L, Ellinghaus D, Hayward C, Navarro P, Ulivi S, Tanaka T, Tester DJ, Chatel S, Gustafsson S, Kumari M, Morris RW, Naluai AT, Padmanabhan S, Kluttig A, Strohmer B, Panayiotou AG, Torres M, Knoflach M, Hubacek JA, Slowikowski K, Raychaudhuri S, Kumar RD, Harris TB, Launer LJ, Shuldiner AR, Alonso A, Bader JS, Ehret G, Huang H, Kao WH, Strait JB, Macfarlane PW, Brown M, Caulfield MJ, Samani NJ, Kronenberg F, Willeit J, Consortium CA, Consortium C, Smith JG, Greiser KH, Meyer Zu Schwabedissen H, Werdan K, Carella M, Zelante L, Heckbert SR, Psaty BM, Rotter JI, Kolcic I, Polasek O, Wright AF, Griffin M, Daly MJ, Dcct/Edic, Arnar DO, Holm H, Thorsteinsdottir U, e MC, Denny JC, Roden DM, Zuvich RL, Emilsson V, Plump AS, Larson MG, O'Donnell CJ, Yin X, Bobbo M, D'Adamo AP, Iorio A, Sinagra G, Carracedo A, Cummings SR, Nalls MA, Jula A, Kontula KK, Marjamaa A, Oikarinen L, Perola M, Porthan K, Erbel R, Hoffmann P, Jockel KH, Kalsch H, Nothen MM, Consortium H, den Hoed M, Loos RJ, Thelle DS, Gieger C, Meitinger T, Perz S, Peters A, Prucha H, Sinner MF, Waldenberger M, de Boer RA, Franke L, van der Vleuten PA, Beckmann BM, Martens E, Bardai A, Hofman N, Wilde AA, Behr ER, Dalageorgou C, Giudicessi JR, Medeiros-Domingo A, Barc J, Kyndt F, Probst V, Ghidoni A, Insolia R, Hamilton RM, Scherer SW, Brandimarto J, Margulies K, Moravec CE, del Greco MF, Fuchsberger C, O'Connell JR, Lee WK, Watt GC, Campbell H, Wild SH, El Mokhtari NE, Frey N, Asselbergs FW, Mateo Leach I, Navis G, van den Berg MP, van Veldhuisen DJ, Kellis M, Krijthe BP, Franco OH, Hofman A, Kors JA, Uitterlinden AG, Witteman JC, Kedenko L, Lamina C, Oostra BA, Abecasis GR, Lakatta EG, Mulas A, Orru M, Schlessinger D, Uda M, Markus MR, Volker U, Snieder H, Spector TD, Arnlov J, Lind L, Sundstrom J, Syvanen AC, Kivimaki M, Kahonen M, Mononen N, Raitakari OT, Viikari JS, Adamkova V, Kiechl S, Brion M, Nicolaides AN, Paulweber B, Haerting J, Dominiczak AF, Nyberg F, Whincup PH, Hingorani AD, Schott JJ, Bezzina CR, Ingelsson E, Ferrucci L, Gasparini P, Wilson JF, Rudan I, Franke A, Muhleisen TW, Pramstaller PP, Lehtimaki TJ, Paterson AD, Parsa A, Liu Y, van Duijn CM, Siscovick DS, Gudnason V, Jamshidi Y, Salomaa V, Felix SB, Sanna S, Ritchie MD, Stricker BH, Stefansson K, Boyer LA, Cappola TP, Olsen JV, Lage K, Schwartz PJ, Kaab S, Chakravarti A, Ackerman MJ, Pfeufer A, de Bakker PI and Newton-Cheh C. Genetic association study of QT interval highlights role for calcium signaling pathways in myocardial repolarization. *Nat Genet*. 2014;46:826-36.  **2.** Sinnott-Armstrong N, Tanigawa Y, Amar D, Mars N, Benner C, Aguirre M, Venkataraman GR, Wainberg M, Ollila HM, Kiiskinen T, Havulinna AS, Pirruccello JP, Qian J, Shcherbina A, Rodriguez F, Assimes TL, Agarwala V, Tibshirani R, Hastie T, Ripatti S, Pritchard JK, Daly MJ and Rivas MA. Genetics of 35 blood and urine biomarkers in the UK Biobank. *Nat Genet*. 2021;53:185-194.  **3.** Xie T, Wang B, Nolte IM, van der Most PJ, Oldehinkel AJ, Hartman CA and Snieder H. Genetic Risk Scores for Complex Disease Traits in Youth. *Circ Genom Precis Med*. 2020;13:e002775.  **4.** Klarin D, Verma SS, Judy R, Dikilitas O, Wolford BN, Paranjpe I, Levin MG, Pan C, Tcheandjieu C, Spin JM, Lynch J, Assimes TL, Åldstedt Nyrønning L, Mattsson E, Edwards TL, Denny J, Larson E, Lee MTM, Carrell D, Zhang Y, Jarvik GP, Gharavi AG, Harley J, Mentch F, Pacheco JA, Hakonarson H, Skogholt AH, Thomas L, Gabrielsen ME, Hveem K, Nielsen JB, Zhou W, Fritsche L, Huang J, Natarajan P, Sun YV, DuVall SL, Rader DJ, Cho K, Chang KM, Wilson PWF, O'Donnell CJ, Kathiresan S, Scali ST, Berceli SA, Willer C, Jones GT, Bown MJ, Nadkarni G, Kullo IJ, Ritchie M, Damrauer SM and Tsao PS. Genetic Architecture of Abdominal Aortic Aneurysm in the Million Veteran Program. *Circulation*. 2020;142:1633-1646.  **5.** Tadros R, Francis C, Xu X, Vermeer AMC, Harper AR, Huurman R, Kelu Bisabu K, Walsh R, Hoorntje ET, Te Rijdt WP, Buchan RJ, van Velzen HG, van Slegtenhorst MA, Vermeulen JM, Offerhaus JA, Bai W, de Marvao A, Lahrouchi N, Beekman L, Karper JC, Veldink JH, Kayvanpour E, Pantazis A, Baksi AJ, Whiffin N, Mazzarotto F, Sloane G, Suzuki H, Schneider-Luftman D, Elliott P, Richard P, Ader F, Villard E, Lichtner P, Meitinger T, Tanck MWT, van Tintelen JP, Thain A, McCarty D, Hegele RA, Roberts JD, Amyot J, Dubé MP, Cadrin-Tourigny J, Giraldeau G, L'Allier PL, Garceau P, Tardif JC, Boekholdt SM, Lumbers RT, Asselbergs FW, Barton PJR, Cook SA, Prasad SK, O'Regan DP, van der Velden J, Verweij KJH, Talajic M, Lettre G, Pinto YM, Meder B, Charron P, de Boer RA, Christiaans I, Michels M, Wilde AAM, Watkins H, Matthews PM, Ware JS and Bezzina CR. Shared genetic pathways contribute to risk of hypertrophic and dilated cardiomyopathies with opposite directions of effect. *Nat Genet*. 2021;53:128-134.  **6.** Khera AV, Chaffin M, Aragam KG, Haas ME, Roselli C, Choi SH, Natarajan P, Lander ES, Lubitz SA, Ellinor PT and Kathiresan S. Genome-wide polygenic scores for common diseases identify individuals with risk equivalent to monogenic mutations. *Nat Genet*. 2018;50:1219-1224.  **7.** Abraham G, Malik R, Yonova-Doing E, Salim A, Wang T, Danesh J, Butterworth AS, Howson JMM, Inouye M and Dichgans M. Genomic risk score offers predictive performance comparable to clinical risk factors for ischaemic stroke. *Nature communications*. 2019;10:5819.  **8.** Elliott J, Bodinier B, Bond TA, Chadeau-Hyam M, Evangelou E, Moons KGM, Dehghan A, Muller DC, Elliott P and Tzoulaki I. Predictive Accuracy of a Polygenic Risk Score-Enhanced Prediction Model vs a Clinical Risk Score for Coronary Artery Disease. *Jama*. 2020;323:636-645.  **9.** Klarin D, Busenkell E, Judy R, Lynch J, Levin M, Haessler J, Aragam K, Chaffin M, Haas M, Lindström S, Assimes TL, Huang J, Min Lee K, Shao Q, Huffman JE, Kabrhel C, Huang Y, Sun YV, Vujkovic M, Saleheen D, Miller DR, Reaven P, DuVall S, Boden WE, Pyarajan S, Reiner AP, Trégouët DA, Henke P, Kooperberg C, Gaziano JM, Concato J, Rader DJ, Cho K, Chang KM, Wilson PWF, Smith NL, O'Donnell CJ, Tsao PS, Kathiresan S, Obi A, Damrauer SM and Natarajan P. Genome-wide association analysis of venous thromboembolism identifies new risk loci and genetic overlap with arterial vascular disease. *Nat Genet*. 2019;51:1574-1579. | | | | |

**Supplemental Figure 1. Probability of diLQTS for QT PGS by Race**

**
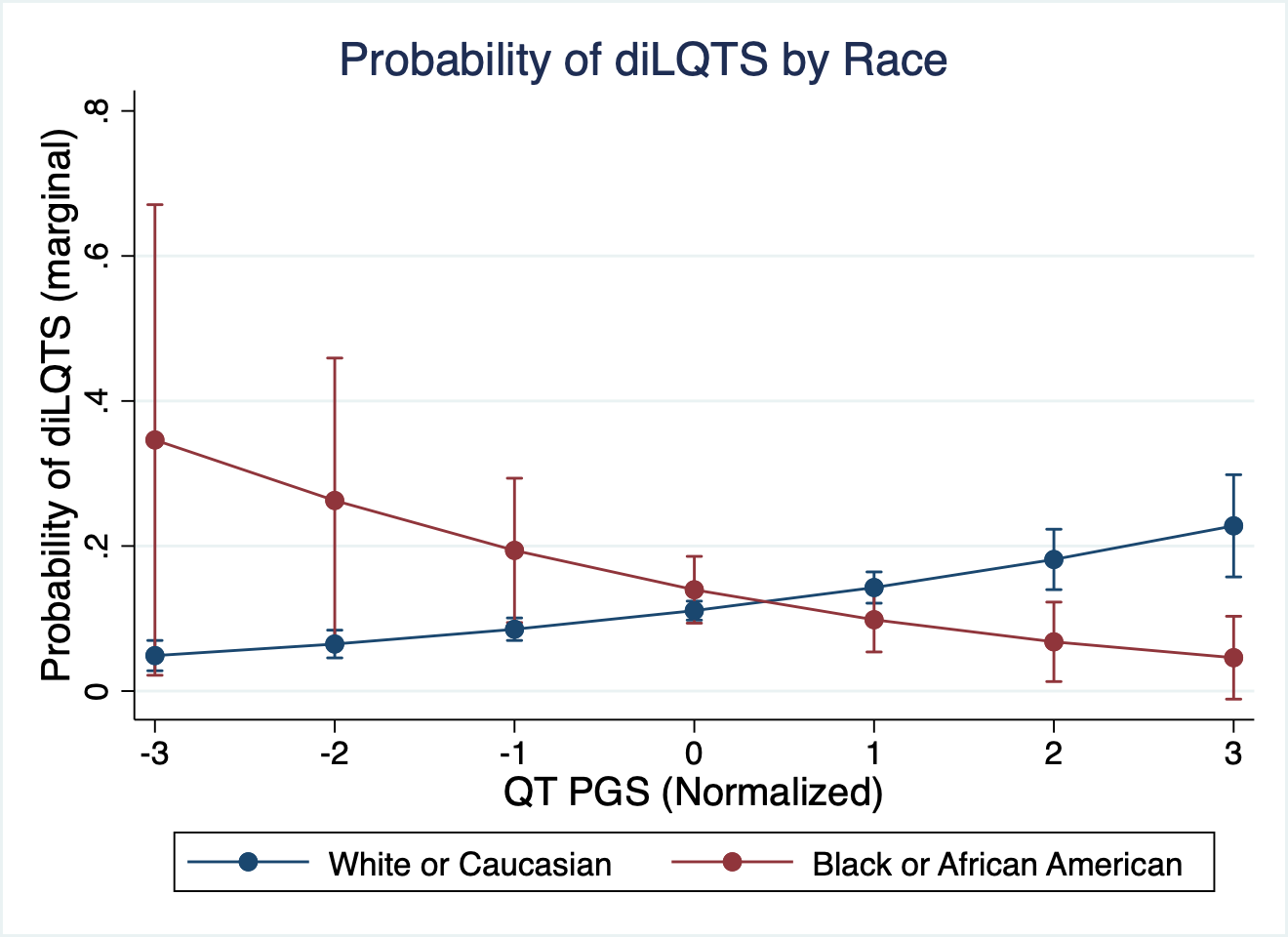
**

**Supplemental Table 2. Demographics by race/ancestry.**

|  | **Caucasian ancestry**  **(N = 1942)** | **African-American ancestry**  **(N = 253)** | **P value** |
| --- | --- | --- | --- |
| **Cases of diLQTS (%)** | 219 (11.3%) | 27 (10.7%) | 0.778 |
| **Age in years (SD)** | 46.4 (15.5) | 54.9 (15.6) | < 0.001 |
| **Female sex (%)** | 1091 (56.2%) | 128 (50.6%) | 0.093 |
| **AF diagnosis (%)** | 356 (18.3%) | 22 (8.7%) | < 0.001 |
| **HF diagnosis (%)** | 305 (15.7%) | 44 (17.4%) | 0.490 |
| **Amiodarone (%)** | 96 (4.9%) | 11 (4.4%) | 0.679 |
| **Dofetilide** | 62 (3.2%) | 0 (0%) | N/A* |
| **Levofloxacin (%)** | 164 (8.4%) | 13 (5.1%) | 0.069 |
| **Propofol (%)** | 97 (5.0%) | 11 (4.4%) | 0.654 |

*Note: No African-American subjects were treated with dofetilide. AF = Atrial fibrillation; HF = Heart failure.

**Supplemental Figure 2. Distribution of QT PGS by Race.**

**
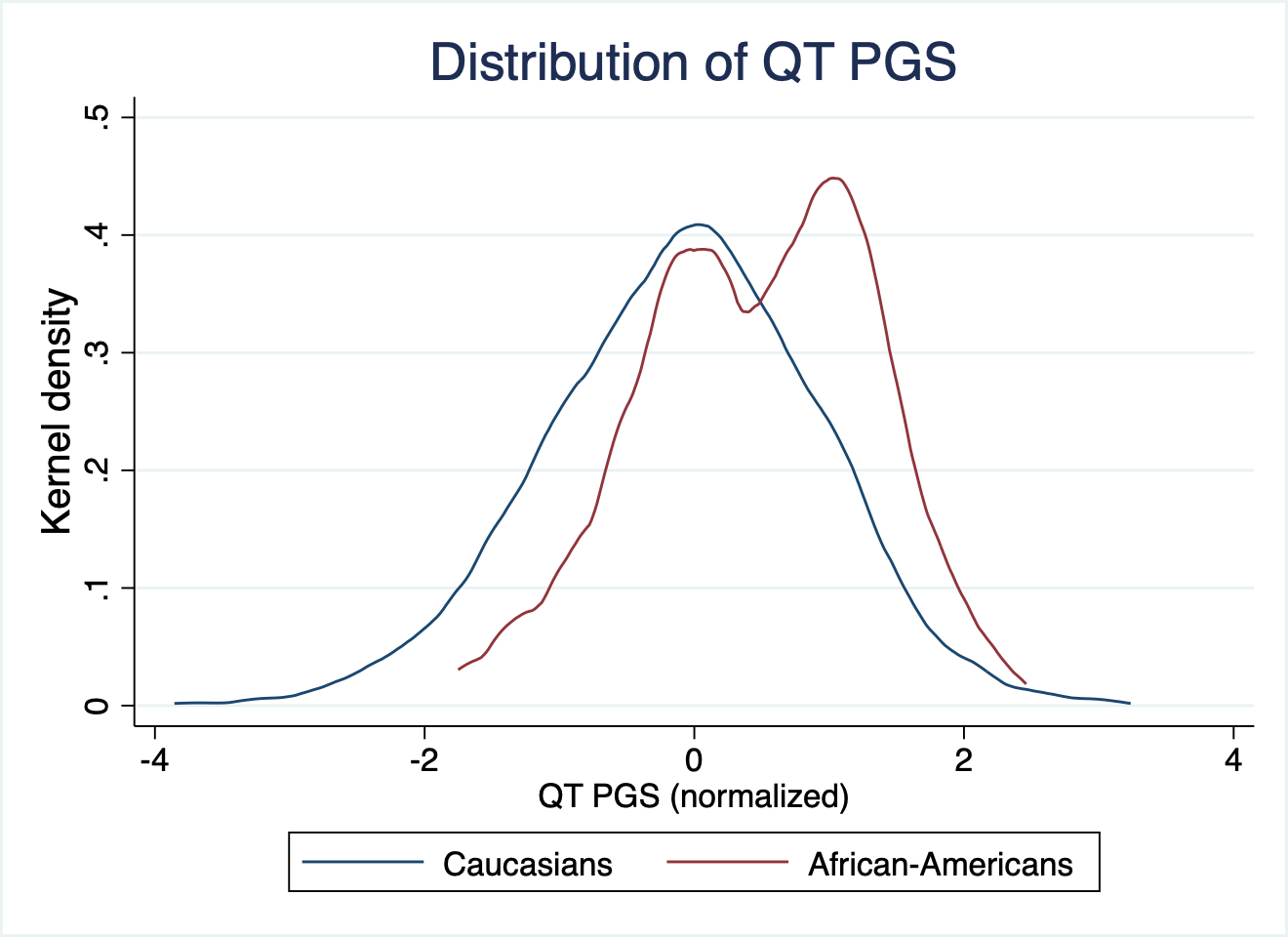
**

**Supplemental Table 3. MIC values for top 50 diagnoses and procedures**

| **Diagnosis Name** | **Code** | **MIC** |
| --- | --- | --- |
| Long QT syndrome | I45.81 | 0.02797087 |
| Cardiogenic shock | R57.0 | 0.01690698 |
| Paroxysmal atrial fibrillation | I48.0 | 0.01485181 |
| 42731:Atrial fibrillation:Atrial fibrillation | 427.31 | 0.01447922 |
| Unspecified atrial fibrillation | I48.91 | 0.01336928 |
| 4280:CHF NOS:Congestive heart failure, unspecified | 428 | 0.01079934 |
| Fluid overload, unspecified | E87.70 | 0.01037194 |
| Persistent atrial fibrillation | I48.1 | 0.01029909 |
| Hypokalemia | E87.6 | 0.01022961 |
| Cardiomyopathy, unspecified | I42.9 | 0.00993387 |
| Ventricular tachycardia | I47.2 | 0.00962243 |
| Acute respiratory failure with hypoxia | J96.01 | 0.00913735 |
| Acute systolic (congestive) heart failure | I50.21 | 0.00909394 |
| Heart failure, unspecified | I50.9 | 0.00734338 |
| 78551:Cardiogenic shock:Cardiogenic shock | 785.51 | 0.00711517 |
| Acute posthemorrhagic anemia | D62 | 0.00678216 |
| Coagulation defect, unspecified | D68.9 | 0.00605528 |
| Unspecified atrial flutter | I48.92 | 0.00596559 |
| 42821:Ac systolic hrt failure:Acute systolic heart failure | 428.21 | 0.00594635 |
| Long term (current) use of anticoagulants | Z79.01 | 0.005791 |
| 4254:Prim cardiomyopathy NEC:Other primary cardiomyopathies | 425.4 | 0.00573829 |
| Acute on chronic systolic (congestive) heart failure | I50.23 | 0.00553179 |
| Thrombocytopenia, unspecified | D69.6 | 0.0055163 |
| Acute kidney failure, unspecified | N17.9 | 0.00529571 |
| Hypertensive heart disease with heart failure | I11.0 | 0.00513147 |
| Dependence on respirator [ventilator] status | Z99.11 | 0.00478056 |
| 27669:Fluid overload NEC:Other fluid overload | 276.69 | 0.00472414 |
| Pleural effusion, not elsewhere classified | J90 | 0.00471793 |
| Shock, unspecified | R57.9 | 0.00471696 |
| 4275:Cardiac arrest:Cardiac arrest | 427.5 | 0.00464291 |
| Hyperglycemia, unspecified | R73.9 | 0.00463212 |
| Acidosis | E87.2 | 0.00462996 |
| Severe sepsis with septic shock | R65.21 | 0.00453273 |
| Other acute postprocedural pain | G89.18 | 0.00452157 |
| Chronic systolic (congestive) heart failure | I50.22 | 0.00440642 |
| Cardiac arrest, cause unspecified | I46.9 | 0.00433264 |
| Hypertensive heart and chronic kidney disease with heart failure and stage 1 through stage 4 chronic kidney disease, or unspecified chronic kidney disease | I13.0 | 0.00432472 |
| 51881:Acute respiratry failure:Acute respiratory failure | 518.81 | 0.00425784 |
| 7906:Abn blood chemistry NEC:Other abnormal blood chemistry | 790.6 | 0.00414818 |
| Nonrheumatic mitral (valve) insufficiency | I34.0 | 0.00410056 |
| Atherosclerotic heart disease of native coronary artery without angina pectoris | I25.10 | 0.00401017 |
| Acute kidney failure with tubular necrosis | N17.0 | 0.0036534 |
| 41010:AMI anterior wall,unspec:Acute myocardial infarction of other anterior wall, episode of care unspecified | 410.1 | 0.00363468 |
| 9095:Lte efct advrs efct drug:Late effect of adverse effect of drug, medicinal or biological substance | 909.5 | 0.00361522 |
| Sick sinus syndrome | I49.5 | 0.00360046 |
| Long term (current) use of aspirin | Z79.82 | 0.00347241 |
| Dilated cardiomyopathy | I42.0 | 0.00343085 |
| ST elevation (STEMI) myocardial infarction involving left anterior descending coronary artery | I21.02 | 0.00342541 |
| Patient room in hospital as the place of occurrence of the external cause | Y92.230 | 0.00341356 |
